# A Gaussian Mixture Model Approach for Estimating and Comparing the Shapes of Distributions of Neuroimaging Data: Diffusion-Measured Aging Effects in Brain White Matter

**DOI:** 10.3389/fpubh.2014.00032

**Published:** 2014-04-14

**Authors:** Namhee Kim, Moonseong Heo, Roman Fleysher, Craig A. Branch, Michael L. Lipton

**Affiliations:** ^1^Department of Radiology, The Gruss Magnetic Resonance Research Center, Albert Einstein College of Medicine, Bronx, NY, USA; ^2^Department of Epidemiology and Population Health, Albert Einstein College of Medicine, Bronx, NY, USA; ^3^Department of Physiology and Biophysics, Albert Einstein College of Medicine, Bronx, NY, USA; ^4^Department of Psychiatry and Behavioral Sciences, Albert Einstein College of Medicine, Bronx, NY, USA; ^5^Dominick P. Purpura Department of Neuroscience, Albert Einstein College of Medicine, Bronx, NY, USA; ^6^Department of Radiology, Montefiore Medical Center, Bronx, NY, USA

**Keywords:** Gaussian mixture model, density function estimation, aging effects, fractional anisotropy, diffusion tensor imaging

## Abstract

Neuroimaging signal intensity measures underlying physiology at each voxel unit. The brain-wide distribution of signal intensities may be used to assess gross brain abnormality. To compare distributions of brain image data between groups, *t*-tests are widely applied. This approach, however, only compares group means and fails to consider the shapes of the distributions. We propose a simple approach for estimating both subject- and group-level density functions based on the framework of Gaussian mixture modeling, with mixture probabilities that are testable between groups. We demonstrate this approach by application to the analysis of fractional anisotropy image data for assessment of aging effects in white matter.

## Introduction

Fractional anisotropy (FA), a scalar measure derived from diffusion tensor imaging (DTI), indexes the degree of anisotropy of water diffusion in brain tissue. In normal white matter (WM), water diffusion is highly constrained to predominantly move parallel to the long axis of axon bundles, or nerve fibers. High FA is thus characteristic of normal WM and may be an indicator of its health. Low FA, on the other hand, can reflect loss of WM microstructural elements in tissues, which normally have high FA and may be an indicator of disease. Low WM FA is associated with demyelinating disease, dementia, traumatic brain injury (TBI), and normal aging.

Inter-subject variability of WM microstructure has been reported with normal subjects as well as patients with various neurodegenerative diseases (1–6). Neurodegeneration is also a feature of normal aging and WM effects of aging disrupt cerebral connectivity, leading to cognitive dysfunction (7, 8). Commonly applied statistical analyses, e.g., *t*-tests at each voxel, may be inherently insensitive to disease pathology due to inter-subject spatial variation (2–4, 9). A whole brain (or whole WM) histogram approach has been used in some studies to address this limitation (10, 11). For example, Benson et al. (10) estimated kurtosis, skewness, peak height, and mean from histograms of WM FA in TBI patients and normals. They aggregated these measures to test for group differences in the shapes of the individuals’ histograms. This analysis demonstrated that the FA distribution of TBI patients exhibited higher kurtosis, higher peak height, and greater skew toward higher FA, but lower mean, compared to those derived from controls. Although this approach used standard statistical summary measures, differences among the shapes of distributions between groups are not easily understood using these summary measures. In addition, these summary measures are not proper for description of multimodal distributions, a special case, which could result from a mixture of multiple distributions (12). Therefore, we propose that estimation and comparison of density functions between groups based on a mixture distribution approach will be more relevant to brain imaging data, which can exhibit unusual distributions.

In this study, we propose a simple approach to estimate subject-level density functions. The technique is based on a Gaussian mixture model (GMM), which assigns subject-specific mixing probabilities to latent underlying Gaussian densities *a posteriori* to characterize an overall distribution of each subject. Estimation of group-level density functions will be based on the estimated subject-level density functions. Differences between groups therefore only depend on the composition of mixture probabilities to underlying Gaussian densities, which lead to an easy and intuitive comparison between groups. For instance, in a simple GMM assuming two Gaussian density components with equal variance constraint, a mixture density function with higher mixing probability to the Gaussian components with lower mean is characterized as a distribution with lower mean and positive skew, while the opposite is characterized as a distribution with higher mean and negative skew. Additionally, the mixture density function with mixing probabilities (1/2, 1/2) is a symmetric distribution with the highest variance in the data. We apply the proposed method to normal control subjects, to examine the effects of aging on the brain-wide distribution of FA.

## General GMM

A general form of a GMM with *m* components can be expressed as follows:

(1) $$f\left(z_{ij};\theta \right)=\sum_{k=1}^{m}\tau \left(k\right)\;\phi_{k}\;\left(z_{ij}\right),$$

where *Z_ij_* is FA measurement of the *j*-th voxel (*j* = 1, …, *N*) observed from the *i*-th subject (*i* = 1, …, *n*). The density function *f* (.) is assumed to be a convex combination of *m* latent Gaussian densities $\left(\phi_{k}\left(Z_{ij}\right)=\phi \left(\frac{Z_{ij}-\mu_{k}}{\sigma_{k}}\right),k=1,\ldots \text{ , }m\right)$ with corresponding *m* mixing probabilities [τ(*k*), *k* = 1, …, *m*], where ϕ(.) is the standard normal density function, and **θ** = (μ*_k_*, σ*_k_*, and τ*_k_, k* = 1, …, *m*). The likelihood function based on model (1) is expressed as follows:

$$\text{L}\left(z;\theta \right)=\prod_{i=1}^{n}\prod_{j=1}^{N}\left[\sum_{k=1}^{m}\tau \left(k\right)\;\phi_{k}\;\left(z_{ij}\right)\right].$$

We assume that the variances are the same across the *m*-Gaussian densities, i.e., σ_1_ = …σ*_m_* = σ, which reduces ϕ*_k_* (*k* = 1, …, *m*) to $\phi_{k}\left(Z_{ij}\right)=\phi \left(\frac{Z_{ij}-\mu_{k}}{\sigma }\right)$ in Eq. 1. The *m* latent Gaussian densities then differ only by their centers. We order the centers of the *m*-Gaussian densities as μ_1_ < … < μ_m_. This parameterization gives easy interpretation of results for comparison of density functions across subgroups. For example, a density function with higher mixing probabilities for low order Gaussian densities will have a smaller mean than that with higher mixing probabilities for higher order Gaussian densities; a density with mixing probability of 1/2 to each of the two Gaussian densities (lowest, highest) will have the largest variance than any other combination of mixture probabilities.

To estimate the parameters **θ** of the general GMM with equal variance constraint, we applied an expectation maximization (EM) algorithm (13); of note, a Bayesian mixture modeling approach (14, 15) is an alternative approach. Specifically, the EM algorithm treats the mixture model in Eq. (1) as an incomplete likelihood function with missing membership information for each observation. In each iteration of the EM algorithm, expectation-step (E-step) computes expectation of complete specification of log-likelihood function with respect to membership values with given data and estimated parameters, and maximization-step (M-step) computes maximum likelihood estimates of parameters specified in the likelihood function with the expected membership values (16). The applied EM algorithm is provided in Table 1. The total number of parameters with equal variance condition for *m*-Gaussian densities is 2*m* [=*m* (means) + *m* − 1 (mixing probabilities) + 1 (variance)]. To determine the optimal number of the latent Gaussian densities, ϕ*_k_*’s (*k* = 1, …, *m*), we used the Akaike information criterion (AIC), which is expressed as 2d−2log  lik(z|θ^)=2d−2∑i=1n∑j=1Nlog∑k=1mτ^ (k) ϕ^k(z). So that the optimal number of Gaussian densities (*m*) is associated with a minimum AIC value.

**Table 1:** **EM algorithm adopted for this study**.

Probability density function	$f\left(z\right)=\sum_{k=1}^{m}\tau \left(k\right)\;\phi_{k}\left(z\right)$
E-step	$\tau_{ij}^{\left(t\right)}\left(k\right)=\;\frac{\tau^{\left(t\right)}\left(k\right)\;\phi_{k}^{\left(t\right)}\;\left(z_{ij}\right)}{\sum_{l=1}^{m}\tau^{\left(t\right)}\left(l\right)\;\phi_{l}^{\left(t\right)}\;\left(z_{ij}\right)}$
M-step	$\mu_{k}^{\left(t+1\right)}=\;\frac{\sum_{i=1}^{n}\sum_{j=1}^{N}z_{ij}\;\tau_{ij}^{\left(t\right)}\left(k\right)}{\sum_{i=1}^{n}\sum_{j=1}^{N}\;\tau_{ij}^{\left(t\right)}\left(k\right)}$
	${\sigma^{2}}^{\left(t+1\right)}=\;\frac{\sum_{i=1}^{n}\sum_{j=1}^{N}\sum_{k=1}^{m}{\left(z_{ij}-\mu_{k}^{\left(t+1\right)}\right)}^{2}\;\tau_{ij}^{\left(t\right)}\left(k\right)}{\sum_{i=1}^{n}\sum_{j=1}^{N}\sum_{k=1}^{m}\tau_{ij}^{\left(t\right)}\left(k\right)}$
	$\tau^{\left(t+1\right)}\left(k\right)=\frac{1}{nN}\sum_{i=1}^{n}\sum_{j=1}^{N}\tau_{ij}^{\left(t\right)}\left(k\right)$

*A variable noted with (*t*) is the estimated value for the variable at the *t*-th iteration*.

### Estimation of subject- and group-level density functions

In this section, we propose a method to estimate subject- and group-level density functions and their associated mixing probabilities. This approach fits the general GMM of Eq. 1 to the full dataset (*Z_ij_, i* = 1, …, *n*; *j* = 1, …, *N*), all voxels from all subjects, and estimates subject- and group-level density functions *a posteriori*.

Under the general GMM, the membership probability of *z_ij_* to *k*-th Gaussian density, denoted by τ*_ij_*(*k*), is obtained based on Bayes theorem as follows:

(2) $$\tau_{ij}\left(k\right)\;=\;\frac{\tau \left(k\right)\;\phi_{k}\left(z_{ij}\right)}{\sum_{l=1}^{m}\tau \left(l\right)\;\phi_{l}\;\left(z_{ij}\right)},$$

which results in $\sum_{k=1}^{m}\tau_{ij}\left(k\right)=1$.

The estimated subject-level density function *f_i_* for the *i*-th subject can be expressed as follows:

(3) $$f_{i}\left(z_{ij};\theta \right)=\sum_{k=1}^{m}\tau_{i}\left(k\right)\;\phi_{k}\;\left(z_{ij}\right),$$

with ϕ*_k_*(*z_ij_*) (*k* = 1, …, *m*) estimated from the general GMM Eq. (1) using all voxel data from all subjects. The parameter τ*_i_*(*k*) in Eq. 3 is the mixing probability of the *k*-th Gaussian density for the *i*-th subject, which we estimate as an average of membership probabilities of *Z_ij_* (*j* = 1, …, *N*) as follows:

$$\tau_{i}\left(k\right)=\frac{1}{N}\sum_{j=1}^{N}\tau_{ij}\left(k\right),$$

where τ*_ij_*(*k*) was estimated based on Eq. 2. Similarly, the estimated group-level density function $f_{g^{*}}$ for the *g*-th group can be expressed as follows:

(4) $$f_{g^{*}}\left(z_{ij}\right)=\sum_{k=1}^{m}\tau_{g^{*}}\left(k\right)\;\phi_{k}\;\left(z_{ij}\right),$$

where $\tau_{g^{*}}\left(k\right)$ in Eq. 4 is the mixing probability of the *k*-th Gaussian density for the *g*-th group, which we estimate as an average of τ*_i_*(*k*) of the subjects in the *g*-th group as follows:

$$\tau_{g^{*}}\left(k\right)=\frac{1}{n_{g}}\sum_{i\;\in \;G_{g}}\tau_{i}\left(k\right)=\frac{1}{n_{g}N}\sum_{i\;\in \;G_{g}}\sum_{j=1}^{N}\tau_{ij}\left(k\right),$$

where *n_g_* is the number of subjects in the *g*-th group.

## Example

### Subjects

The Albert Einstein College of Medicine Institutional Review Board (IRB) approved and monitored this study. Twenty-eight normal subjects without history of head injury, cardiovascular or cerebrovascular disease, diabetes, or neurological or psychiatric disease were recruited between August 2006 and May 2010 through advertisements. Demographic data for the 28 normal subjects are summarized in Table 2.

**Table 2:** **Subjects’ demographic characteristics**.

	Gender	Number of subjects	Mean years of education (SD)	Minimum years of education	Maximum years of education
All ages	All genders	28	13.6 (1.6)	12	18
	Female	16	13.9 (1.8)	12	18
	Male	12	13.2 (1.3)	12	16
20–29	All	7	14.1 (1.7)	12	16
	Female	4	14.3 (1.7)	12	16
	Male	3	14.0 (2.0)	12	16
30–39	All	7	12.9 (1.0)	12	14
	Female	4	12.8 (1.0)	12	14
	Male	3	13.0 (1.0)	12	14
40–49	All	7	13.4 (1.6)	12	16
	Female	4	14.3 (1.7)	12	16
	Male	3	12.3 (0.6)	12	13
50–59	All	7	13.9 (2.0)	12	18
	Female	4	14.3 (2.6)	12	18
	Male	3	13.3 (1.2)	12	14

### Diffusion tensor image acquisition and image data preprocessing

Imaging was performed using a 3.0 T MRI scanner (Achieva; Philips Medical Systems, Best, The Netherlands) with an eight-channel head coil (Sense Head Coil; Philips Medical Systems). T1-weighted whole-head structural imaging was performed using sagittal three-dimensional magnetization-prepared rapid acquisition gradient echo (MP-RAGE; TR/TE = 9.9/4.6 ms; field of view, 240 mm^2^; matrix, 240 × 240; and section thickness, 1 mm). T2-weighted whole-head imaging was performed using axial two-dimensional turbo spin-echo (TR/TE = 4000/100 ms; field of view, 240 mm^2^; matrix, 384 × 512; and section thickness, 4.5 mm). DTI was performed using single-shot echo-planar imaging (TR/TE = 3800/88 ms; field of view, 240 mm^2^; matrix, 112 × 89; section thickness, 4.5 mm; independent diffusion sensitizing directions, 32; and *b* = 800 s/mm^2^). The images were preprocessed as described previously (4, 9).

### Demonstration of the proposed methods using example FA image data

Fractional anisotropy datasets were normalized prior to fitting the proposed models. The normalization was necessary because it is well-known that FA is heterogeneous across brain regions; for example, higher FA is characteristic of deep WM such as the corpus callosum, and lower FA is typical of peripheral WM. Specifically, we normalized each FA measurement *x_ij_* by $z_{ij}=\frac{x_{ij}-{\bar{x}}_{j}}{s_{j}}$ with mean $\left({\bar{x}}_{j}\right)$ and standard deviation (*s_j_*) of *n* subjects (*i* = 1, …, *n*) at each voxel (*j* = 1, …, *N*).

Subjects were divided into four age groups: 20–29, 30–39, 40–49, and 50–59 years. Each age group consisted of seven subjects (four women and three men). Age groups were matched for years of education, differing by no more than 2 years; no significant difference in years of education was detected among age groups (*p* = 0.76). Two Gaussian densities were required for the approach based on the AIC.

**Figure 1:**
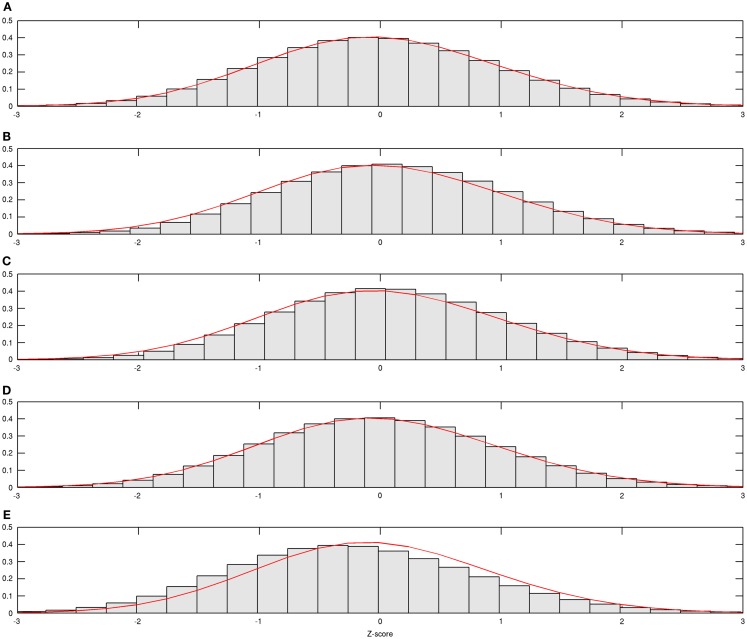
**Estimated age group-wise density functions**. Estimated density function for all and each age group is demonstrated; **(A)** from all subjects, **(B)** from subjects aged 20–29, **(C)** from subjects aged 30–39, **(D)** from subjects aged 40–49, and **(E)** from subjects aged 50–59.

**Figure 2:**
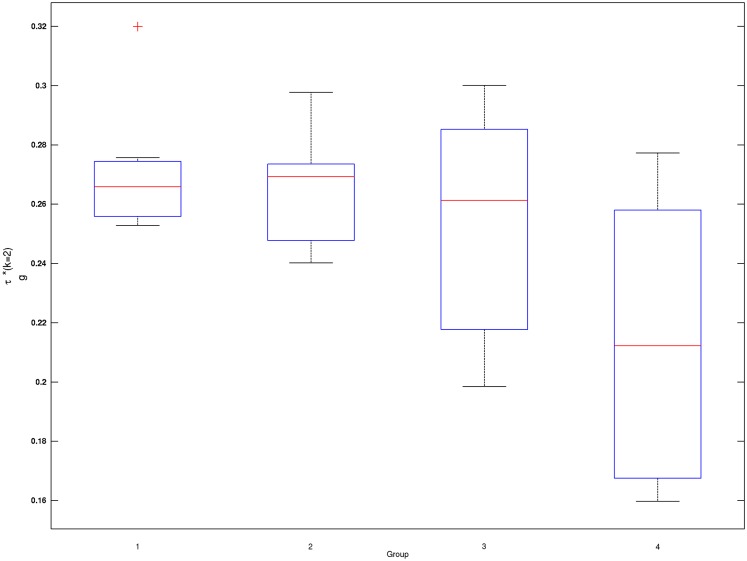
**Box plots of subject-wise mixing probabilities by each age group (*G* = 1, 2, 3, 4)**. Subject-level estimated mixing probability to the second Gaussian density [τ_i_(*k* = 2), *i* = 1, …, *n*] is demonstrated for each of the age groups (*G* = 1, 2, 3, 4). Outliers in each box plot, marked with red + signs, are defined as values that are more than 1.5 times the interquartile range away from the top or bottom of the box.

In Figure 1, estimated density functions across the entire control group (*n* = 28) and each of the four age groups are presented $\left(f\left(z\right),f_{g^{*}}\left(z\right),g=\text{1},\text{2},\text{3},\text{4}\right)$. Inference on the difference in shapes of FA distribution across age groups was performed by estimating mixing probabilities from the proposed estimation method; these results are shown in Table 3 and Figure 2. We order estimated Gaussian densities (ϕ*_k_*) by their centers, in ascending manner; thus, we identify ϕ_1_ as the Gaussian density with the lowest center.

**Table 3:** **Estimated mixing probabilities for each age group**.

Age	$\tau_{g^{*}}$ (*k* = 1) (s.e.)	$\tau_{g^{*}}$ (*k* = 2) (s.e.)
All	0.7488 (0.0076)	0.2512 (0.0076)
20–29	0.7277 (0.0086)	0.2723 (0.0086)
30–39	0.7337 (0.0075)	0.2663 (0.0075)
40–49	0.7481 (0.0150)	0.2519 (0.0150)
50–59	0.7873 (0.0184)^a^	0.2127 (0.0184)^a^

*^a^Two-sided *p*-value < 0.05. Each Gaussian density is specified as μ_1_ = −0.26, μ_2_ = 0.78, and σ = 0.87*.

Mixing probabilities of the two Gaussian densities, *k* = 1 and *k* = 2, show opposite patterns of change across age groups. As age increases the mixing probability of the first Gaussian density (*k* = 1) increases while that of the second Gaussian density (*k* = 2) decreases. Box plots describing distributions of subject-level estimated mixing probabilities to the second Gaussian density [τ_i_(*k* = 2), *i* = 1, …, *n*] are provided for all age groups in Figure 2. While mixing probabilities to the second Gaussian density (*k* = 2) were lower for older age group in that lower mean FA for older age group, higher between-subject variance is noted in Figure 2. A Kruskal–Wallis test was performed to compare subject-level mixing probabilities between Group = 1 and Group = *g* (*g* = 2, 3, 4). A significant difference in the mixing probability was found between Groups 1 (20–29 years) and 4 (50–59 years) with *p* = 0.048 with degrees of freedom (1, 12). This significantly lower mean mixing probability to the Gaussian density with a greater mean implies that FA declines significantly over the age of 50 years. This pattern also implies lower intra-subject variance in FA distribution for the age group (50–59 years) because of higher mixing probability to the first Gaussian density (*k* = 1). While all age groups showed positively skewed FA distributions (Table 3), the youngest group, aged 20–29 years, showed the greatest skewness to the right. However, the Kruskal–Wallis test, which compares all four groups was not significant (*p* = 0.141, df = 3, 24).

## Discussion

The proposed approach for subgroup density estimation with GMM allows group comparison based on shape parameters represented by the relative mixing probabilities of the latent Gaussian densities. Since Gaussian densities are estimated based on the measurements from all voxels of all subjects, estimated individual densities will differ only by their own mixing probabilities. Individual densities are thus characterized by comparing the estimated mixing probabilities. Although kernel density estimation (KDE) (17) is a widely applied statistical approach for density estimation, a density function estimated by KDE does not provide shape parameters for comparison between groups because the parameter determining the shape of a density function by KDE is only the bandwidth for the chosen kernel function. In contrast, mixing probabilities estimated in the present study enable comparison of shapes between groups, which we have demonstrated with a real FA data set.

The GMM approach proposed herein was designed to discriminate density functions across different subgroups. This approach implicitly assumes that the density function derived from all voxels will represent a mixture of at least two Gaussian components. Testing for differences in the shapes of the density functions from different groups is then possible by testing the means of mixing rates assigned to *m*-Gaussian densities (where *m* ≥ 2) between groups. However, if one Gaussian density function (i.e., *m* = 1) is sufficient to fit the entire distribution, discrimination of density functions between different subgroups is not available with the proposed approach. Additionally, the proposed GMM is a highly parsimonious approach in that it does not incorporate any subject- or group-specific shape parameters in the likelihood function; further improvement may be achieved by their inclusion.

A GMM with unequal variance assumption resulted in little difference in fitting performance compared with the proposed approach with equal variance assumption (data not shown). Nevertheless, application of non-Gaussian mixtures could be employed, study of which is beyond the scope of the present paper and should serve as a future study.

Initial application of the method to human FA datasets revealed that the distribution of FA differs significantly between subjects aged 20–29 years and those aged 50–59 years. Age-related change in FA distribution was found in mean, variance (intra-subject, between-subject), and skew. Since aging is a feature of neurodegeneration, this finding may have an implication to other neurodegenerative diseases, e.g., dementia and Alzheimer’s disease. Since the present demonstration is based on a small sample, further examinations with larger samples are warranted to fully characterize the utility of this approach and the age effects it has revealed.

## Conflict of Interest Statement

The authors declare that the research was conducted in the absence of any commercial or financial relationships that could be construed as a potential conflict of interest.

## References

[1] Thiebaut de Schotten M Ffytche DH Bizzi A Dell’Acqua F Allin M Walshe M Atlasing location, asymmetry and inter-subject variability of white matter tracts in the human brain with MR diffusion tractography. Neuroimage (2011) 54(1):49–59 10.1016/j.neuroimage.2010.07.055 20682348

[2] Muller HP Unrath A Riecker A Pinkhardt EH Ludolph AC Kassubek J Intersubject variability in the analysis of diffusion tensor images at the group level: fractional anisotropy mapping and fiber tracking techniques. Magn Reson Imaging (2009) 27(3):324–34 10.1016/j.mri.2008.07.003 18701228

[3] Kou Z Wu Z Tong KA Holshouser B Benson RR Hu J The role of advanced MR imaging findings as biomarkers of traumatic brain injury. J Head Trauma Rehabil (2010) 25(4):267–82 10.1097/HTR.0b013e3181e54793 20611045

[4] Lipton ML Kim N Park YK Hulkower MB Gardin TM Shifteh K Robust detection of traumatic axonal injury in individual mild traumatic brain injury patients: intersubject variation, change over time and bidirectional changes in anisotropy. Brain Imaging Behav (2012) 6(2):329–42 10.1007/s11682-012-9175-2 22684769

[5] Rosenbaum SB Lipton ML Embracing chaos: the scope and importance of clinical and pathological heterogeneity in mTBI. Brain Imaging Behav (2012) 6(2):255–82 10.1007/s11682-012-9162-7 22549452

[6] Yan H Wang H Wang YH Zhang YM Volumetric magnetic resonance imaging classification for Alzheimer’s disease based on kernel density estimation of local features. Chin Med J (Engl) (2013) 126(9):1654–60 10.3760/cma.j.issn.0366-6999.20122683 23652046

[7] Kochunov P Thompson PM Lancaster JL Bartzokis G Smith S Coyle T Relationship between white matter fractional anisotropy and other indices of cerebral health in normal aging: tract-based spatial statistics study of aging. Neuroimage (2007) 35(2):478–87 10.1016/j.neuroimage.2006.12.021 17292629

[8] Walhovd KB Fjell AM Reinvang I Lundervold A Dale AM Eilertsen DE Effects of age on volumes of cortex, white matter and subcortical structures. Neurobiol Aging (2005) 26(9):1261–70 10.1016/j.neurobiolaging.2005.05.020 16005549

[9] Kim N Branch CA Kim M Lipton ML Whole brain approaches for identification of microstructural abnormalities in individual patients: comparison of techniques applied to mild traumatic brain injury. PLoS One (2013) 8(3):e59382 10.1371/journal.pone.0059382 23555665 PMC3608654

[10] Benson RR Meda SA Vasudevan S Kou Z Govindarajan KA Hanks RA Global white matter analysis of diffusion tensor images is predictive of injury severity in traumatic brain injury. J Neurotrauma (2007) 24(3):446–59 10.1089/neu.2006.0153 17402851

[11] Lipton ML Gellella E Lo C Gold T Ardekani BA Shifteh K Multifocal white matter ultrastructural abnormalities in mild traumatic brain injury with cognitive disability: a voxel-wise analysis of diffusion tensor imaging. J Neurotrauma (2008) 25(11):1335–42 10.1089/neu.2008.0547 19061376

[12] Molas M Lesaffre E Finite mixture models with fixed weights applied to growth data. Biom Lett (2012) 49(2):103–19 10.2478/bile-2013-0008

[13] Dempster P Laird NM Rubin DB Maximum likelihood from incomplete data via the EM algorithm. J Roy Stat Soc B (1977) 39:1–38

[14] Diebolt J Robert CP Estimation of finite mixture distributions through Bayesian sampling. J Roy Stat Soc B Met (1994) 56(2):363–75 10.3168/jds.2010-3470 20965363

[15] Tanner MA Wong HW The calculation of posterior distributions by data augmentation. J Am Stat Assoc (1987) 82(398):528–40 10.2307/2289463

[16] McLachlan G Peel D Finite Mixture Models: Wiley Series in Probability and Mathematical Statistics. John Wiley & Sons, Inc (2000). 419 p.

[17] Gu C Smoothing Spline ANOVA Models. New York: Springer (2002).

